# Thyroid Nodules in Pediatrics: Which Ones Can Be Left Alone, Which Ones Must be Investigated, When and How

**DOI:** 10.4274/Jcrpe.853

**Published:** 2013-03-01

**Authors:** Andrea Corrias, Alessandro Mussa

**Affiliations:** 1 University of Torino, Regina Margherita Children’s Hospital, Department of Pediatric Endocrinology and Diabetology, Torino, Italy

**Keywords:** Thyroid nodules, thyroid cancer, Pediatrics, children

## Abstract

Thyroid nodules are less frequent in childhood than in adulthood, but are more often malignant. Recent estimates suggest that up to 25% of thyroid nodules in children are malignant, therefore, a more aggressive approach is recommended. In this review, we suggest an approach based on a first-step clinical, laboratory, and sonographic evaluation. A history of irradiation of the neck, cranium or upper thorax, previous thyroid diseases or thyroid neoplasms in the family should alert clinicians as being associated with a greater likelihood of malignant nodules. Signs or symptoms of hyperthyroidism and dysmorphic features should be carefully considered during the physical examination. Palpable firm lymph nodes, found in some 70% of cases, are the most significant clinical finding in children with malignant nodules. Although the routine determination of calcitonin levels is not uniformly practiced, it can help recognize sporadic or familial medullary thyroid neoplasms. Blood thyroid stimulating hormone, free thyroxine, and free triiodothyronine determinations (the latter in case of symptoms of hyperthyroidism) are aimed at identifying the few hyperthyroid patients, for whom the next step should be scintiscan. Hyperthyroid patients usually disclose an increased uptake, and a diagnosis of toxic adenoma is commonly made. Cases with normal thyroid function or hypothyroidism (which is usually subclinical) should be evaluated by fine-needle aspiration biopsy (FNAB). In eu/hypo-thyroid patients, scintiscan provides poor diagnostic information and should not be routinely employed. Thyroid ultrasonography is used to select cases for FNAB. Although ultrasound cannot reliably discriminate between benign and malignant lesions, it does provide an index of suspicion. Sonographic features that increase the likelihood of malignancy are microcalcifications, lymph node alterations, nodule growth under levothyroxine treatment, and increased intranodular vascularization demonstrated by color Doppler. There is growing evidence that elastography may provide further information on nodule characteristics. FNAB is indicated in all cases with a likelihood of malignancy. FNAB has a diagnostic accuracy of approximately 90% and is used in selection of patients which require surgery. Recently, histological markers and elastography have been introduced to increase the specificity of FNAB and ultrasound, respectively. The pitfall in FNAB cytology is the follicular cytology, in which it is not possible to distinguish between adenoma and carcinoma and therefore thyroidectomy is advised.

**Conflict of interest:**None declared.

## INTRODUCTION

**Epidemiology**

Nodule prevalence in adults has been estimated to range from 2 to 6% by palpation, from 19 to 35% by sonography, and from 8 to 65% in postmortem examinations (1). Much less is known about the prevalence of thyroid nodules in childhood and adolescence. Valuable series have been reported in recent years, but almost all available data regarding thyroid nodules and cancer in pediatrics are retrospective. Based on a large epidemiological study in the USA in 1975, it was estimated that 1.79% of children have palpable nodules (2,3). More recently, ultrasound studies in pediatrics have revealed a prevalence ranging from 0.2 to 5.1% (4,5). 

Current estimates suggest that up to 25% of thyroid nodules in children are malignant, compared to 5% in adults (4,6). In a recent review by Niedziela (4) in which data from 16 papers published between 1960 and 2004 were analyzed, the overall incidence was 26.4%, ranging from 9.2 % to 50% (7). However, these data should be considered with caution, as they are hardly comparable. The diagnostic procedures employed to detect thyroid nodules were not homogeneous in these studies. The early studies were conducted by physical examination (i.e. thyroid palpation), which is commonly considered as a subjective method. Later, routine ultrasound evaluation revealed a consistently higher prevalence of thyroid nodules (8). It should be noted that it is not rare to find nodules larger than 2 cm that can only be revealed by a sonographic investigation (8), and although a physical examination is quite effective in detecting the nodules localized in the isthmus or in the anterior surface, it is much less accurate in detecting nodules localized in the upper pole of the gland, even when they are large. The second epidemiological difference between adult and pediatric thyroid nodules is that in pediatrics, a prevalence of females has not been reported as is typical of adulthood (9).

Endocrine cancers are very rare in children, with thyroid cancer being the most common one constituting 0.5% to 3% of all childhood malignancies (9). The Surveillance, Epidemiology and End Results (SEER) Cancer Statistics Review of the U.S. National Institute of Health reported across 1975-2006 an incidence of 1 per million for 5-9 year-old children, 5 per million in 10-14 year-olds, and 18 per million in 15-19 year-olds. The incidence in females is higher, with a 3:1 ratio before 15 years and 6:1 in the 15-19 year age group (1,9). 

Thyroid carcinomas in childhood are almost always well-differentiated. Recently, a multicentric study conducted on 120 pediatric patients with a thyroid nodule not associated with risk factors such as autoimmune thyroid diseases or radiotherapy revealed a 16% occurrence of thyroid carcinoma, with 12% papillary, 2.5% follicular, and 1.7% medullary histotypes (3). However, although they are usually slow-growing tumors, a prompt diagnosis is recommended, because greater tumor size, distant spread, and greater atypia are all factors associated with a worse prognosis and increased mortality (4). Benign tumors account for approximately 10% of all nodules (3), and among these, of prominent importance for its clinical relevance and therapeutic implications is the toxic adenoma (Plummer adenoma).

Once diagnosed, pediatric thyroid nodules require immediate attention and, for the most precise diagnostic definition, an accurate multistep workup, including anamnestic, objective, laboratory, and instrumental evaluations, is necessary (Table 1). 

**Causes, Predisposing Factor, and Associated Diseases**

Thyroid nodular disease encompasses a wide spectrum of disorders ranging from isolated thyroid nodules to multinodular goiter. It can present as primary thyroid disease or within a pre-existing thyroid pathology, either autoimmune or not. It can be characterized by a clinical picture suggestive of hyperthyroidism or be clinically silent and is increasingly encountered as an incidental finding in thyroid or neck ultrasound performed because of a positive family history or some other condition (4). 

Familial occurrence for thyroid neoplasms is striking and well-documented for medullary thyroid cancer (MTC), and it is also frequently present in other histotypes (9). Preexisting thyroid diseases represent a further risk factor. Cases of cancer have also been described in congenital hypothyroidism due to dyshormonogenesis, in iodide organification defects, in ectopy, as well as in thyroid hemiagenesis and thyroglossal duct cysts, usually of follicular histotypes. Studies on thyroid autoimmune diseases in the adult population investigated their relationship with thyroid cancer, in particular with papillary histotypes. The prevalence of thyroid cancer among patients with thyroid autoimmunity is a matter of some controversy. After Dailey et al (10) reported 35 cases of autoimmune thyroiditis among 288 patients with malignant thyroid disease postulating that the disorder was a precancerous lesion, other investigators sought an association between autoimmune thyroiditis and thyroid cancer and reported a prevalence ranging from 1% to 32% (11). Differences in patient selection most likely contributed to this variability that was also associated with geographic and ethnic heterogeneity in both thyroid autoimmunity and thyroid cancer prevalence. Recently, in a study on 365 pediatric patients with autoimmune thyroiditis, we reported that thyroid cancer was present in approximately 3% of patients, occurring in 9.6% of the subset with thyroid nodules (11). In this latter study, only taking into account nodules with diameters over 1 cm, the occurrence of thyroid cancer was 20%, an estimate that closely parallels that reported for nodules in non-autoimmune thyroiditis patients. This consideration highlights the importance of a careful workup of thyroid nodules with diameters of 1 cm or larger, even when occurring in patients with autoimmune thyroid disease. Indeed, a diameter greater than 1 cm is widely employed in clinical practice as the cut-off for clinical alert and subsequent investigations, although from our experience, exceptions and other risk factors should also be carefully considered.

Exposure to ionizing radiations is another well-known risk factor for thyroid cancer, especially for papillary carcinoma. Children’s thyroid gland is much more sensitive to the carcinogenic effects of ionizing radiation than that of adults. Lower doses than those observed for most other radiation-induced cancers may induce thyroid carcinogenesis (to the order of 0.10 Gy). High doses (>30 Gy) result in a lowered risk of thyroid cancer, probably due to cell killing (12). From a historical viewpoint, the high incidence of thyroid cancers in the 1950s and 1960s following the extensive use of low-dose radiotherapy for benign pathologies of the head, neck, and chest is worth mentioning. In one of the earliest reports on this subject, Duffy et al (7) showed how 35% of the pediatric patients with thyroid cancer had previously been irradiated for thymus enlargement. Pediatric thyroid cancer had an increasing incidence from the beginning of the last century until peaking in the late 1950s and then declined, reflecting the increasing awareness of the cancer-causing effect of radiation (13,14). 

A great deal of our knowledge about radiation effects on thyroid carcinogenesis arises from evidence from exposure to radioactive isotopes in the fallout from Chernobyl. The observed increase in the incidence of thyroid carcinoma was first noticed in children, and the risk of developing thyroid cancer was strongly correlated with young age at exposition and the estimated radiation dose (15). Almost exclusively, a papillary histotype developed (16) that exhibited aggressive growth and early metastasis (17). 

Radiation is also used in the treatment of several childhood malignancies and there is increasing evidence that patients submitted to such treatment in childhood might be prone to developing cancerous and non-cancerous thyroid diseases in youth (18). After a first tumor in childhood, thyroid cancers are most likely to arise 6-7 years or more after the primary cancer, and the radiation-associated risk for thyroid cancer remains elevated for at least 20 years (19,20). In radiotherapy, the risk of thyroid cancer increases parallel to radiation dosages of up to 20-29 Gy, but then falls at higher doses (12). With these premises and considering that an early diagnosis might improve the outcome, it has recently been suggested to screen by ultrasound the population of childhood cancer survivors who had previously undergone radiotherapy involving the head, neck or upper thorax (18). Furthermore, it should be considered that there is increasing evidence that chemotherapy might also be a causative factor (21).

**Genetics and Inheritance of Medullary and Nonmedullary Thyroid Neoplasms**

In recent decades, molecular studies have elucidated a number of critical genetic pathways associated with the development of thyroid tumors. In papillary thyroid carcinoma, genetic alterations such as RET/TRK rearrangements or BRAF and RAS mutations usually lead to signaling derangements in the mitogen-activated protein kinase pathway. The first mechanism is largely prevalent in childhood (22). Compared to sporadic tumors, thyroid carcinomas arising in individuals with a history of radiation exposure show a very high prevalence of gene rearrangements. Although the most common is RET/PTC, BRAF/AKAP9 is also found predominantly in radiation-induced papillary thyroid carcinoma (23). In spite of a prognostic significance which is still debated, a correlation between RET/PTC gene rearrangements and tumor aggressiveness has been postulated (24). In follicular carcinomas, genetic alterations commonly consist of PAX8-PPAR gamma translocations or RAS mutations, whereas CTNNB1 and p53 mutations have been implicated in the development of anaplastic carcinomas (22).

Furthermore, of paramount importance for pediatricians is the knowledge of a number of somatic genetic conditions presenting or associated with thyroid nodules and cancer. Inherited thyroid neoplasms may be the preeminent feature of the disorder in nonsyndromic familial non MTC encompassing pure familial papillary thyroid carcinoma with or without papillary renal cell carcinoma, and of the papillary thyroid carcinoma with multinodular goiter. These patients most likely have autosomal dominant mutations with incomplete penetrance and disclose multifocal papillary carcinoma. 

Pediatricians should also be aware of a number of genetic syndromes presenting with thyroid nodules, most of which are overgrowth, hamartomatous, and cancer predisposition syndromes with a preponderance of non-thyroidal tumors (Table 2). PTEN hamartoma tumor syndrome is a heterogeneous group of disorders encompassing Cowden, Bannayan-Riley-Ruvalcaba, Proteus, and Proteus-like syndromes caused by different germline inactivating mutations of the PTEN tumor suppressor gene. Patients with Cowden syndrome are prone to develop both benign and malignant tumors in a variety of tissues, the most frequently affected being the uterus, breast, bowel, and thyroid. On the contrary, Bannayan-Riley-Ruvalcaba syndrome usually manifests in pediatrics with macrocranium, multiple lipomatosis, retarded neuropsychomotor development, and thyroid adenomas possibly associated with autoimmune thyroiditis. Typical of males is the presence of hyperpigmented macules on the glans penis. Thyroid nodules are usually follicular with hyperplastic multinodular goiter, and thyroid carcinomas are encountered in 50-67% and 5-10% of cases, respectively (25). 

domAn increased risk of thyroid nodules and cancer is well-documented in intestinal polyposis syndromes. Familial adenomatous polyposis and its variant Gardner’s syndrome (having mandibular osteomas, fibromas, and sebaceous cysts as additional features) are caused by mutations in the APC gene. Peutz-Jeghers syndrome is a separate autosomal inant multiple gastrointestinal hamartomatous polyposis syndrome characterized by melanotic macules on the lips and oral mucosa, also carrying an increased risk of thyroid cancer besides other neoplasms. 

Carney complex is an autosomal dominant disease characterized by skin, breast, and cardiac myxomas, skin and mucosal hyperpigmentation (lentiginosis), and endocrine gland neoplasias. These latter include a variable association of pituitary adenomas, Sertoli and Leydig cell tumors, pigmented nodular adrenal disease, and thyroid multinodular disease. Thyroid nodules are more frequently follicular and histologically benign, although 10-15% of them are a differentiated cancer, with most cases being caused by mutations in the PRKAR1-α tumor-suppressor gene.

Among inherited conditions, McCune-Albright syndrome is a similar sporadic condition that is also characterized by multiple endocrine and non-endocrine neoplasias. The cause is the post-zygotic gain-of-function mutations in GNAS1, encoding for the α-subunit of many G-coupled receptors. Although the disorder is clinically characterized by the classic triad of polyostotic fibrous dysplasia, café-au-lait skin spots, and peripheral precocious puberty, the mosaic distribution of the mutation in various tissues indicates the possibility of occurrence of various other endocrinological anomalies such as thyrotoxicosis, hyperpituitarism, and Cushing syndrome. In McCune-Albright syndrome, a cystic or nodular feature coupled with autonomous thyroid function rather than malignancies is the main feature (26). Hyperthyroidism is commonly due to multinodular toxic goiter (27) and is one of the rare causes of neonatal hyperthyroidism.

MTC originates from calcitonin-producing C cells derived from the neural crest and accounts for approximately 5% of all thyroid tumors (28). The familial form accounts for 20- 25% of cases and is either one of the features of multiple endocrine neoplasia (MEN) 2A or 2B, or a pure familia MTC syndrome. MEN2 syndromes are characterized by the co-occurrence of MTC and pheochromocytoma. In type A, the additional features are parathyroid gland tumors with hyperparathyroidism, whereas the association of mucosal neuromas, intestinal ganglioneuromatosis, and marfanoid habitus is typical of type B (29). In both cases, patients have specific mutations in the RET proto-oncogene. C-cell hyperplasia is the precursor of cancer in these inheritable syndromes. Activating mutations of the RET proto-oncogene are present in approximately 95% of inheritable cases (30). These mutations lead to RET constitutive signaling and consequent C-cell hyperplasia with calcitonin hypersecretion and subsequent malignant transformation. Therefore, calcitonin can be employed as a “screening” tool for medullary cancer in thyroid nodules. Familial MTC is commonly associated with mutations at codons 618 and 620 and with noncysteine mutations at codons 768 and 804 (31,32). MEN 2A is caused by a germline mutation in exons 10 and 11 of the RET gene (32). In MEN 2B, familial occurrence is not so strong as mutations frequently occur de novo. As the penetrance for MTC is almost complete in both MEN 2A and MEN 2B, prophylactic thyroidectomy is advised. Its timing varies according to the RET mutation based on genotype-phenotype studies (31,33). Calcitonin also has a critical role in the follow-up of patients submitted to thyroidectomy.

**Objective Examination**

Hard and fixed nodules, characteristics suggestive of malignancy, can be detected by palpation. Once the nodule is detected, either clinically or sonographically, the objective examination is mainly aimed at 1) evaluating the presence of associated lymph node enlargement, 2) ascertaining or excluding a clinical picture suggestive of hyperthyroidism, and 3) assessing the presence of local compression symptoms. The clinician is therefore expected to look for dysphagia, dysphonia, discomfort, or shortness of breath to assess local compression, and for tachycardia, increased differential pressure, hyperhydrosis, diarrhea, weight loss, increased appetite, heat intolerance, tremors, or exophthalmia for hyperthyroidism (34). 

The finding of lymph node enlargement is of crucial importance. Hard and firm lymph nodes are commonly considered highly suggestive of thyroid malignancy. This is of utmost importance in the pediatric age group as lymph node involvement is strikingly more common that in adult patients (6,35) and has been estimated to occur in up to 78.9% of cases (36,37) although this characteristic is not associated with a worse prognosis. Taking into consideration various findings such as the number of nodules, lymph node enlargement, faint uptake on scintiscan, hypoechogenicity, and the cytology result, the presence of a clinically detectable firm lymph node enlargement ranked second after cytology in predicting the likelihood of malignancy in pediatric thyroid nodules (36). 

A physical examination should be complete and extensive, including auxologic assessment and should also aim to detect signs and dysmorphisms typical of syndromes associated with thyroid nodules. Particular attention should be placed on detecting signs and symptoms of MEN2 syndromes.

**Laboratory Assessment**

The main purposes of laboratory assays are to evaluate thyroid function and to assess calcitonin secretion. Determinations of thyroid-stimulating hormone (TSH), free thyroxine (fT4), and free triiodothyronine (fT3) levels allow distinguishing between euthyroidism, hypothyroidism, and hyperthyroidism - thyroid states which represent approximately 90%, 5%, and 5% of cases, respectively. Indeed, most thyroid nodules occur in euthyroidism, in patients with no systemic symptoms. The same is also true for nodules occurring in hypothyroid patients in whom hypothyroidism is commonly subclinical. Recently, there has been much interest for TSH determinations in nodule diagnosis (38,39,40,41,42,43,44). TSH values in the upper reference range (i.e. in the upper half of normality) seem to correlate with the likelihood of malignancy. Although interesting, this finding necessitates further confirmation in the pediatric age group, and its usefulness in a clinical setting needs further validation from the scientific community. 

On the other hand, hyperthyroidism is commonly clinically relevant and associated with a larger nodule size. Although nodules in hypothyroid patients have the same diagnostic management as euthyroid ones, in cases with hyperthyroidism, a further diagnostic approach is needed. In patients with euthyroidism or hypothyroidism the next step should be fine-needle aspiration biopsy (FNAB). Scintiscan offers very little informative value and may even provide misleading information (3) showing hyperfunctioning areas in the thyroid tissue. On the other hand, in hyperthyroid patients, the key diagnostic step is scintiscan as it allows a visual distinction of the area of hyperfunctioning thyroid tissue from the residual parenchyma that can be either hyperfunctioning (a “hot” nodule) or normally functioning (a “warm” nodule). As hyperthyroid nodules are commonly follicular and only rarely harbor malignancies, FNAB is often considered superfluous. However, one should bear in mind that a papillary carcinoma has been found in approximately 5% of these nodules (4). Toxic adenoma has been poorly characterized in the pediatric literature as being rather uncommon in this age group, with its estimated prevalence ranging from 0.7% (45) to 5% (3) of thyroid nodules. Toxic adenoma quite often causes compression symptoms due to the greater nodule size than in euthyroid patients (3). Hyperthyroidism is commonly caused by a toxic adenoma with a prevalence ranging from 0.7 to 5% (3,7). In these latter cases, scintiscan usually discloses a “hot” pattern with the remnant thyroid tissue being silent. FNAB does not offer much information in these cases, and surgical intervention is required for an optimal management (3,6,46).

Calcitonin is the most sensitive diagnostic tool for MTC. Its role is particularly important in the screening for MTC, allowing a better outcome that is probably due to the significant lower stage of the disease at diagnosis. However, still its routine use as a first-step evaluation of a newly discovered nodule is debated: some authors do not suggest a routine determination (47), but most do (48). Determination of calcitonin levels has been recommended since 2006 by the European Thyroid Association (ETA) (49) but not by the American Thyroid Association (ATA) (46) which emphasizes the unsolved question of its cost-effectiveness (50). A recent collaborative work from the American Association of Clinical Endocrinologists, the Associazione Medici Endocrinologi, and the European Thyroid Association (AACE/AME/ETA) Task Force on Thyroid Nodules also questioned the systematic assessment of calcitonin levels in patients with thyroid nodules (51). Nevertheless, routine assessment of calcitonin levels is mandatory in patients with a suspicion of familial MTC, MEN2, and in cases showing a cytology suggestive of medullary neoplasm. Our experience suggests a systematic evaluation of calcitonin levels in pediatric patients. 

The main limitation in systematic calcitonin determination lies in the difficulties in establishing reliable cut-off values, because calcitonin concentration physiologically increases with age and weight, differs according to sex, and may result in false positive cases due to its secretion in neuroendocrine disorders, in lung/pancreas cancer, nephropathy, hypergastrinemia, thyroid autoimmunity. Alcohol consumption, smoking, sepsis, and positivity for heterophilic anti-calcitonin antibodies also affect calcitonin levels. In cases of increased calcitonin levels, confirmation by a repeat determination or by performing a stimulation test (calcitonin determination 2, 5, and 15 minutes after pentagastrin 0.5 μg/Kg i.v. bolus) has been suggested (52) in order to increase specificity. This confirmatory test is necessary due to the frequent false positive results (neuroendocrine tumors, another cancer, renal failure, pancreatitis, thyroid diseases, etc). There is agreement about a cut-off value of 100 pg/mL that is certainly indicative of MTC (52). 

In some cases, determination of thyroglobulin levels in the washout fluid of neck lymph nodes is of particular note as thyroglobulin is a sensitive and specific marker of well-differentiated thyroid cancers. This method, previously employed for the early detection of cervical metastases following thyroidectomy and radioiodine therapy, allows an early diagnosis of lymph node metastases before surgery and is mostly employed in cases with small thyroid nodules but with enlarged lymph nodes that are highly suggestive of malignancy (52,53,54). Assessment of calcitonin level in the washout fluid from FNAB of cervical lymph nodes can also be used as a potentially highly reliable diagnostic procedure to identify primary and recurrent/metastatic MTC (52,54,55). 

**Sonography **

Thyroid ultrasound is the first-line screening imaging tool to detect thyroid nodules (56). Its tremendous advantages led to its role at the forefront in the diagnosis and management of thyroid nodules as it is cheap, rapid, radiation-free, non-invasive, and easily available. However, the disadvantages of this method include its being operator-dependent. The patient has to be examined in the supine position, with the neck extended and a small pad should be placed under the shoulders to provide a better extension of the neck. For neck lumps, sonography helps define the tissue pertinence of the nodule. Physicians should be aware that a number of non-thyroidal conditions (such as abscesses, thyroglossal duct cysts, ectopic thymus, lymphatic or vascular malformations, and tumors) may present with a neck lump and mimic a thyroid nodule. The role of sonography, however, is not limited to the confirmation of a thyroid nodule. Sonography is fundamental in assessing the number of nodules in cases in which only one is palpable as well as in assessing the dimensions of the thyroid gland (both data are useful in cases with a multinodular goiter). Also worthy of mention is its role in guiding the FNAB and in monitoring the lymph nodes and remnant thyroid tissue status in thyroidectomized patients.

Most importantly, sonography allows a first-line screening in order to select patients whose nodules have suspicious characteristics and who thus need further evaluations (56,57,58). In such cases, color Doppler sonography usually provides more detailed characteristics of the nodule compared to 2-mode ultrasound. However, it is fundamental to point out that sonographic evaluation alone cannot provide a definitive diagnosis of a benign or malignant nodule, and that further evaluation and follow-up are always needed. Several nodule characteristics have been described as being more commonly indicative of a benign or a malignant disease. Hypoechogenicity with respect to the normal surrounding thyroid tissue is the sonographic feature that has been most closely associated with malignancies (4,46). However, although it is true that the ultrasound pattern of a malignant nodule is frequently homogeneously hypoechoic, it is also true that the same pattern is also demonstrable in many benign nodules. Hypoechogenicity, thus, seems to be a sensitive but not a specific parameter to rely on in the diagnostic algorithm of pediatric thyroid nodules. Other features that point to existence of a malignancy are undefined margins, internal microcalcifications, and high intranodular vascular flow or tortuous internal vessels assessed by color Doppler (4). Some reports also point out that solitary nodules, a subcapsular localization, and a heterogeneous ultrasound pattern are potential indicators of an increased likelihood of cancer (59). An increase in nodule size during the ultrasound follow-up, particularly during a trial with suppressive or sub-suppressive levothyroxine therapy, has also been advocated as an index of malignancy (11,60). 

Sonographic features of associated lymph nodes also potentially increase the likelihood of malignancy, thus suggesting further diagnostic investigation. The ratio between the longitudinal and transversal axes of the lymph nodes below 1.5 (normally greater than 2), rounded profile of a lymph node, absence of hilum, presence of eccentric cortical thickening, nonhomogeneous pattern, and the increased vascular flow are other parameters commonly included as indicators of malignancy (3,4,6,46,61,62,63,64).

**Fine-Needle Aspiration Biopsy **

In the last 30 years, FNAB has become a cornerstone in the evaluation of solitary thyroid nodules, cysts, and dominant nodules within multinodular goiters in adults. Fewer data are available in pediatrics (11,30,65,66,67) given the consistently lower occurrence of the disease. Eight studies dealing with this issue recently (3,11,36,68) estimated its diagnostic accuracy as ranging from 75 to 95%, a value approximating that reported for adults (69,70,71). 

Historically, the safety of this procedure in pediatrics was questioned, arguing its discomfort, limited usefulness, and the high rate of side-effects such as papillary endothelial hyperplasia, hemorrhage, vascular proliferation, vascular thrombosis, fibrosis, cystic change, infarction, and abscesses (71,72). However, more recent evidence has shown that most pediatric patients who underwent FNAB did not develop any complications (36,59,73). Nevertheless, the prominent questions are whether FNAB is the most reliable procedure to detect malignancies and to decide on which cases require surgical treatment, and whether this approach compares favorably with conventional clinical, laboratory, and imaging studies in detecting which patients should undergo surgery. FNAB showed the highest sensitivity, specificity, and accuracy among other diagnostic investigations (36).

There is general agreement on performing FNAB in euthyroid and hypothyroid patients with palpable nodules and in those with nodule diameters greater than 1 cm and with clear sonographic findings indicating a suspicion of malignancy. However, this approach might be questionable in several other situations, such as in non-palpable nodules, multinodular goiter, small nodules, or cystic lesions. Furthermore, another issue is to reach a consensus on cases in which FNAB should be repeated. As far as non-palpable nodules are concerned, some authors opt for FNAB whenever a nodule is accidentally revealed by ultrasound (36). However, this approach might be considered excessively aggressive, also taking into consideration that the papillary histotype (the most frequent one in pediatrics) is commonly a slow-growing tumor with a very indolent course even after local and pulmonary metastasization (74). Some researchers recommend FNAB only in palpable nodules based on epidemiological studies that reported rare occurrences of thyroid cancer in non-palpable nodules. However, these reports are of interest for adult patients since malignancy rate in adulthood is consistently lower than that in pediatrics (51) and fail to consider that in childhood, malignancies have been described irrespective of nodule size (3).

FNAB is commonly recommended for nodules with diameters over 1 cm or for nodules growing in size, particularly nodules which show growth under levo-T4 treatment (75,76), as mentioned above. However, FNAB should also be taken into consideration in nodules smaller than 1 cm (6,11,77), and the diagnostic approach should be particularly aggressive in the presence of clinical or sonographic risk factors such as external radiation for other malignancies of the head, the neck, and upper thorax, a family history of thyroid cancer, rapid nodule growth, presence of sonographic characteristics suggestive of malignancy, or when a suspicious and persistent cervical lymph node enlargement is detected. 

Although malignancies more often manifest as solitary nodules, multinodular thyroid disease seems to carry a comparable risk for malignant neoplasms (3,77,78). Obviously, in such cases, all suspicious nodules should undergo cytological evaluation. Also, as papillary cancer has been reported to occur in 5 to 14% of cystic lesions (59,79), the cystic nature of a nodule should not be considered a reassuring finding and should be submitted to cytology. 

Another issue is the timing of FNAB, and, in particular, to decide on the need for a repeat sample. Most clinicians consider a repeat FNAB appropriate when the previous sampling was non-diagnostic (“undetermined”) (46), and especially in case of highly suspicious clinical or sonographic findings (80). Indications for FNAB are based on a risk-based approach, starting from cystic nodules (in which the FNAB is debated) to cases with several clinical and sonographic risk factors, in which FNAB is mandatory (51). It is worth mentioning the findings of a study reporting papillary cancer in 1.3% of patients with previous benign FNAB results and who underwent annual repeat FNABs for 2-12 years (81). By discriminating malignant nodules, FNAB can avoid a significant number of unnecessary surgical interventions. However, it has been estimated that in up to 20% of thyroid nodules, FNAB cannot provide clear diagnostic indications. These cases are commonly referred to as having a “suspicious cytology”, a category that includes follicular neoplasms (hyperplastic nodules, follicular adenoma, follicular carcinoma, and a follicular variant of papillary carcinoma) (82,83) and Hurthle cell lesions (84). Based on cytology, FNAB specimens are categorized in 5 degrees of risk (TYR1-5) (85). It is well proven that only 20% of cases in this subset are histologically diagnosed with a malignancy, while the remaining 80% undergo a potentially superfluous or untimely surgical intervention. In the attempt to address this diagnostic pitfall, a number of researchers looked for additional tools to resolve this issue, such as elastography, molecular histological markers, and, more recently, 18-fluorodeoxyglucose positron emission tomography/computed tomography (18-FDG PET/CT).

Elastography has recently been introduced in clinical practice to distinguish benign and malignant thyroid nodules. This technique, which is also employed in mammary and hepatic nodular diseases, investigates the stiffness of solid nodules by analyzing the speed of an elastic wave passing through the tissues. The principle it relies on is the increased stiffness of malignant nodules as they are usually firmer than the surrounding tissues. To date, only a few studies have evaluated its use in thyroid nodular disease (45), but some results are encouraging further research and elastography might hold great potential as a new tool in the diagnosis of thyroid cancer, especially in nodules with undetermined cytology, thus allowing a reduction in the number of FNABs performed in up to 60% of cases (51,63,64). However, researchers’ views are conflicting, and more recent papers underline that elastography seems not to be superior to standard ultrasound and that further work is needed in this research area (86,87).

As regards the reliability of immunocytochemistry to differentiate benign and malignant thyroid diseases from a cytological smear (88,89), we report a list (Table 3) of markers and predictors for differentiated thyroid cancer. Among them, galectin-3, HBME-1, cytokeratin-19, CD44v6, and telomerase (90,91,92) are currently considered to be more reliable in discriminating thyroid carcinoma. Obviously, calcitonin also has to be considered as a reliable marker of medullary carcinoma. However, the main limitation of this approach is that none of these markers alone entirely fulfill the clinical diagnostic needs, but rather a complete panel of these markers should be employed to perform a sufficiently reliable diagnostic process. Furthermore, most of these markers have been studied in an adult population, and very few data are available concerning pediatric patients (46). 

Most recently, the literature focused on the use of molecular markers (mutational testing) in an attempt to improve the diagnostics of the malignant thyroid nodule. Several genes, which are known to be almost invariably connected with the proliferation and progression of cellular cycle, have been reported as mutated in malignant thyroid nodules. The most relevant example is the consistent finding of mutations in BRAF in cells from papillary thyroid cancer, which is the commonest in pediatrics. Other relevant mutations found involve the RAS and RET/PTC genes (92). Although further studies are needed to refine these results, the molecular approach seems promising as concerns its cost-effectiveness (93,94).

Finally, 18-FDG PET/CT has also been proposed as a useful tool to differentiate benign and malignant nodules in cases of suspicious FNAB results. Data concerning its employment are scanty and contradictory (95) and do not allow as yet to draw conclusions on the usefulness of this expensive tool. 

**Figure 1:**
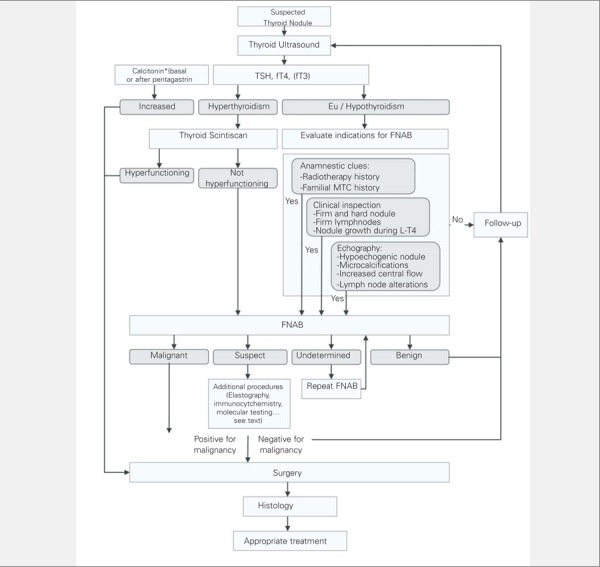
Diagnostic algorithm for pediatric thyroid nodules. A multi-step approach is proposed encompassing a first step with clinicalevaluation, thyroid ultrasound and laboratory assays and a second step including scintiscan in case of hyperthyroidism, or an evaluation forFNAB in case of eu/hypothyroidism* Calcitonin systematic dosage is not universally accepted (see text)FNAB: fine-needle aspiration biopsy, fT4: free thyroxine, fT3: free triiodothyronine, MTC: medullary thyroid cancer, L-T4: levo T4 TSH: thyroid-stimulating hormone

In the light of these considerations, a reasonably acceptable diagnostic algorithm, displayed in Figure 1, can be proposed for thyroid nodules with a diameter of at least 1 cm. As far as smaller nodules are concerned, FNAB should only be considered in selected cases with multiple clues pointing to a malignant lesion. The role of elastography in nodules with undetermined cytology still remains to be better defined in pediatrics, and the employment of this tool in the diagnosis, although promising, needs further verification.

**Conclusions **

Approximately 1 in 4–5 thyroid nodules in pediatrics are malignant. Histotypes are almost constantly well-differentiated. The diagnostic work-up is complex and aims at progressively identifying those nodules with the highest risk for malignancy. A simplified diagnostic algorithm is proposed in Figure 1. We suggest an approach based on a first-step clinical, sonographic, and laboratory evaluations. A history of radiotherapy and family history of thyroid cancer should alert clinicians. The detection of hard palpable nodules or firm lymph node enlargement strongly suggests further evaluation. Although a thyroid sonography cannot consistently discriminate between benign and malignant nodules, it does provide precious information in selecting which patients to be evaluated by FNAB. Hypoechogenicity, microcalcifications, specific lymph node alterations, and increased intranodular vascular flow are all findings indicating a high suspicion for malignancy. Moreover, ultrasound imaging can identify clinically unapparent nodules that can have a likelihood of malignancy comparable to palpable nodules. Determination of blood TSH, fT4, and fT3 levels aims to identify hyperthyroid patients. In hyperthyroid patients, scintiscan represents the next logical diagnostic step, and increased uptake on scintiscans is present in these cases. In toxic adenoma, histology is almost always benign, nevertheless, surgery is commonly required to counteract the hyperthyroidism. Otherwise, in the much more common case of euthyroidism or hypothyroidism, FNAB is indicated as it is the key tool to select which patients should undergo surgery and has around 90% diagnostic accuracy. Undetermined and follicular cytology are the two pitfalls of FNAB. The recent introduction of elastography and the increasingly employed panel of molecular and metabolic markers may increase accuracy in the latter cases.

**Acknowledgements **

The authors are grateful to Prof. Fabio Orlandi from the University of Turin for his precious contribution and help with the review of data concerning the employment of the molecular markers on FNAB cytology, and to Dr. Enrico Brignardello, from the Transition Unit for Childhood Cancer Survivors of the San Giovanni Battista Hospital, Turin, for his valuable suggestions and contribution on matter of the relationship between irradiation and thyroid cancer. We are also grateful to Mr Andrew Martin Garvey for editorial assistance.

## Figures and Tables

**Table 1:**
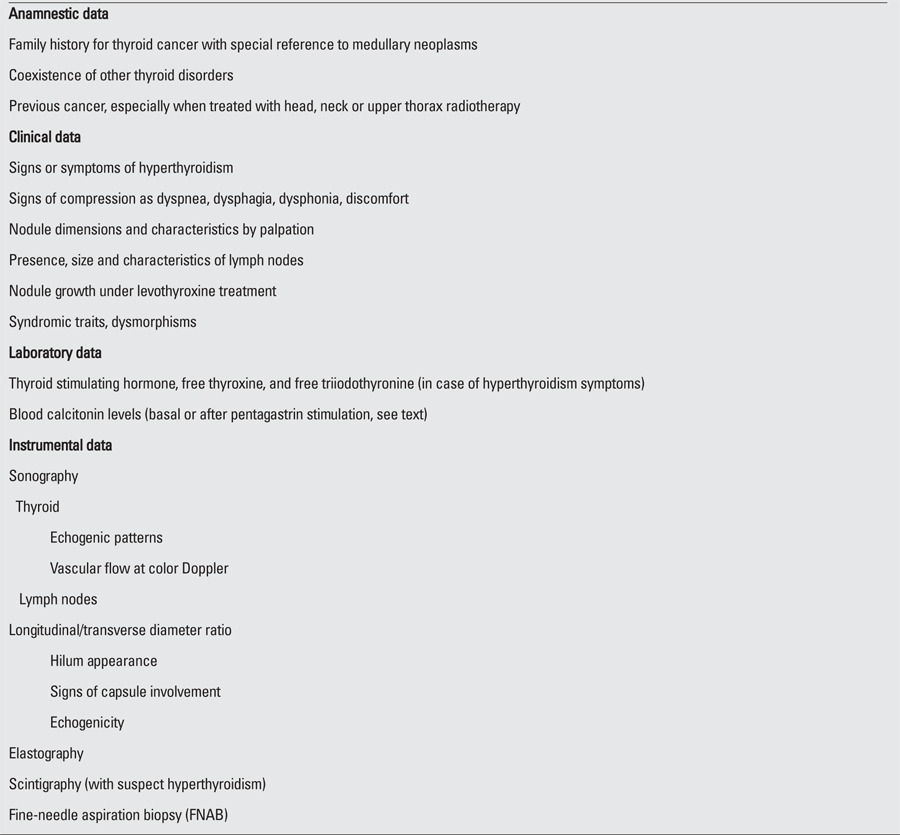
Anamnestic, clinical, laboratory, and instrumental data to be taken into account in the diagnostic approach to pediatric thyroid nodules

**Table 2:**
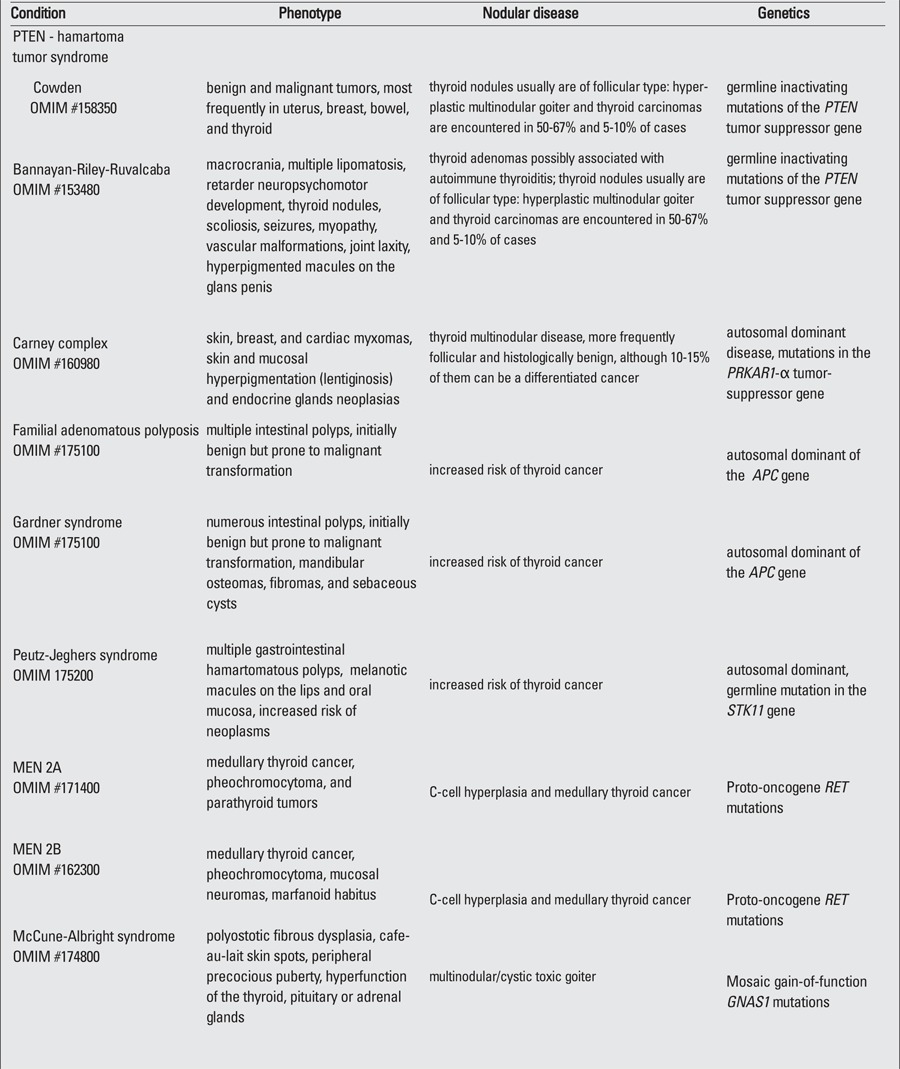
Syndromes and inheritable conditions associated with thyroid nodules and cancer

**Table 3:**
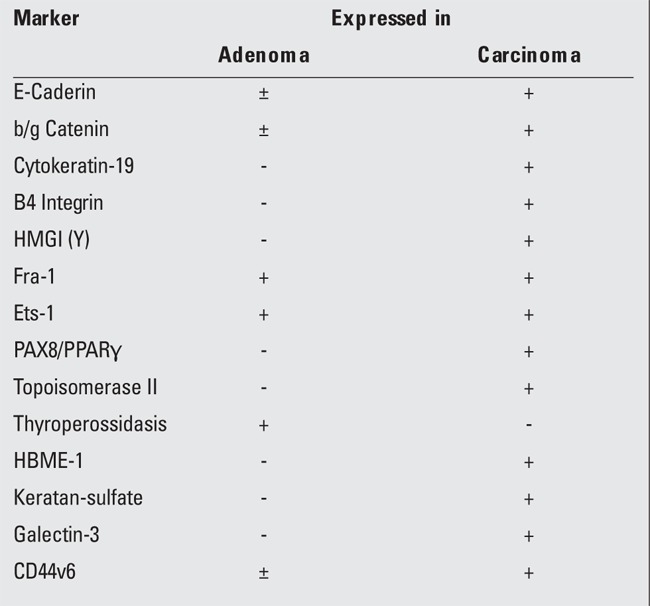
Molecular markers and predictors in differentiated thyroidcarcinoma diagnosed by fine-needle aspiration biopsy (FNAB) cytology

## References

[1] Dean DS, Gharib H (2008). Epidemiology of thyroid nodules. Best Pract Res Clin Endocrinol Metab.

[2] Rallison ML, Dobyns BM, Keating FR Jr, Rall JE, Tyler FH (1975). Thyroid nodularity in children. JAMA.

[3] 3. Corrias A, Mussa A, Baronio F, Arrigo T, Salerno M, Segni M, Vigone MC, Gastaldi R, Zirilli G, Tuli G, Beccaria L, Iughetti L, Einaudi S, Weber G, De Luca F (2010). Diagnostic features of thyroid nodules in pediatrics. Arch Pediatr Adolesc Med.

[4] Niedziela M (2006). Pathogenesis, diagnosis and management of thyroid nodules in children. Endocr Relat Cancer.

[5] 5. Aghini-Lombardi F, Antonangeli L, Martino E, Vitti P, Maccherini D, Leoli F, Rago T, Grasso L, Valeriano R, Balestrieri A, Pinchera A (1999). The spectrum of thyroid disorders in an iodine-deficient community:the Pescopagano survey. J Clin Endocrinol Metab.

[6] Dinauer CA, Breuer C, Rivkees SA (2008). Differentiated thyroid cancer in children:diagnosis and management. Curr Opin Oncol.

[7] Duffy BJ Jr, Fitzgerald PJ (1950). Thyroid cancer in childhood and adolescence; a report on 28 cases. Cancer.

[8] Wang C, Crapo LM (1997). The epidemiology of thyroid disease and implications for screening. Endocrinol Metab Clin North Am.

[9] Halac I, Zimmerman D (2005). Thyroid nodules and cancers in children. Endocrinol Metab Clin North Am.

[10] Dailey ME, Lindsay S, Skahen R (1955). Relation of thyroid neoplasms to Hashimoto disease of the thyroid gland. AMA Arch Surg.

[11] Corrias A, Cassio A, Weber G, Mussa A, Wasniewska M, Rapa A, Gastaldi R, Einaudi S, Baronio F, Vigone MC, Messina MF, Bal M, Bona G (2008). Thyroid nodules and cancer in children and adolescents affected by autoimmune thyroiditis. Arch Pediatr Adolesc Med.

[12] Sigurdson AJ, Ronckers CM, Mertens AC, Stovall M, Smith SA, Liu Y, Berkow RL, Hammond S, Neglia JP, Meadows AT, Sklar CA, Robison LL, Inskip PD (2005). Primary thyroid cancer after a first tumors in childhood the Childhood Cancer Survivor Study): a nested case control study. Lancet.

[13] Scott MD, Crawford JD (1976). Solitary thyroid nodules in childhood: is the incidence of thyroid carcinoma declining?. Pediatrics.

[14] White AK, Smith RJ (1986). Thyroid nodules in children. Otolaryngol Head Neck Surg.

[15] Rabes HM, Demidchik EP, Sidorow JD, Lengfelder E, Beimfohr C, Hoelzel D, Klugbauer S (2000). Pattern of radiation-induced RET and NTRK1 rearrangements in 191 post-chernobyl papillary thyroid carcinomas: biological, phenotypic, and clinical implications. Clin Cancer Res.

[16] Nikiforov Y, Gnepp DR. (1994). Pediatric thyroid cancer after the Chernobyl disaster. Pathomorphological study of 84 cases (1991-1992) from the Republic of Belarus.. Cancer.

[17] Pacini F, Vorontsova T, Demidchik EP, Molinaro E, Agate L, Romei C, Shavrova E, Cherstvoy ED, Ivashkevitch Y, Kuchinskaya E, Schlumberger M, Ronga G, Filesi M, Pinchera A (1997). Post-Chernobyl thyroid carcinoma in Belarus children and adolescents: comparison with naturally occurring thyroid carcinoma in Italy and France. J Clin Endocrinol Metab.

[18] Brignardello E, Corrias A, Isolato G, Palestini N, Fagioli F, Boccuzzi G (2008). Ultrasound screening for thyroid carcinoma in childhood cancer survivors: a case series. J Clin Endocrinol Metab.

[19] Ron E, Lubin JH, Shore RE, Mabuchi K, Modan B, Pottern LM, Schneider AB, Tucker MA, Boice JD Jr (1995). Thyroid cancer after exposure to external radiation: a pooled analysis of seven studies. Radiat Res.

[20] Acharya S, Sarafoglou K, LaQuaglia M, Lindsley S, Gerald W, Wollner N, Tan C, Sklar C (2003). Thyroid neoplasms after therapeutic radiation for malignancies during childhood or adolescence. Cancer.

[21] Cohen A, Rovelli A, Merlo DF, Lint MT, Lanino E, Bresters D, Ceppi M, Bocchini V, Tichelli A, Socie G (2007). Risk for secondary thyroid carcinoma after hematopoietic stem-cell transplantation: an EBMT Late Effects Working Party Study. J Clin Oncol.

[22] Yamashita S, Saenko V (2007). Mechanisms of Disease: molecular genetics of childhood thyroid cancers. Nat Clin Pract Endocrinol Metab.

[23] Gandhi M, Evdokimova V, Nikiforov YE (2010). Mechanisms of chromosomal rearrangements in solid tumours: the model of papillary thyroid carcinoma. Mol Cell Endocrinol.

[24] Collins BJ, Chiappetta G, Schneider AB, Santoro M, Pentimalli F, Fogelfeld L, Gierlowski T, Shore-Freedman E, Jaffe G, Fusco A (2002). RET expression in papillary thyroid cancer from patients irradiated in childhood for benign conditions. J Clin Endocrinol Metab.

[25] Harach HR, Soubeyran I, Brown A, Bonneau D, Longy M (1999). Thyroid pathologic findings in patients with Cowden disease. Ann Diagn Pathol.

[26] Congedo V, Celi FS (2007). Thyroid disease in patients with McCune-Albright syndrome. Pediatr Endocrinol Rev.

[27] Hamilton CR Jr, Maloof F (1973). Unusual types of hyperthyroidism. Medicine (Baltimore).

[28] Massoll N, Mazzaferri EL (2004). Diagnosis and management of medullary thyroid carcinoma. Clin Lab Med.

[29] Raue F, Frank-Raue K (2007). Multiple endocrine neoplasia type 2: 2007 update. Horm Res.

[30] Kapila K, Pathan SK, George SS, Haji BE, Das DK, Qadan LR (2010). Fine needle aspiration cytology of the thyroid in children and adolescents: experience with 792 aspirates. Acta Cytol.

[31] Eng C, Clayton D, Schuffenecker I, Lenoir G, Cote G, Gagel RF, Amstel HK, Lips CJ, Nishisho I, Takai SI, Marsh DJ, Robinson BG, Frank-Raue K, Raue F, Xue F, Noll WW, Romei C, Pacini F, Fink M, Niederle B, Zedenius J, Nordenskjold M, Komminoth P, Hendy GN, Mulligan LM, et al (1996). The relationship between specific RET proto-oncogene mutations and disease phenotype in multiple endocrine neoplasia type 2. International RET mutation consortium analysis. JAMA.

[32] Learoyd DL, Marsh DJ, Richardson AL, Twigg SM, Delbridge L, Robinson BG (1997). Genetic testing for familial cancer. Consequences of RET proto-oncogene mutation analysis in multiple endocrine neoplasia, type 2. Arch Surg.

[33] Fialkowski EA, Moley JF (2006). Current approaches to medullary thyroid carcinoma, sporadic and familial. J Surg Oncol.

[34] Mazzaferri EL (1993). Management of a solitary thyroid nodule. N Engl J Med.

[35] Brink JS, Heerden JA, McIver B, Salomao DR, Farley DR, Grant CS, Thompson GB, Zimmerman D, Hay ID (2000). Papillary thyroid cancer with pulmonary metastases in children: long-term prognosis. Surgery.

[36] Corrias A, Einaudi S, Chiorboli E, Weber G, Crino A, Andreo M, Cesaretti G, Sanctis L, Messina MF, Segni M, Cicchetti M, Vigone M, Pasquino AM, Spera S, Luca F, Mussa GC, Bona G (2001). Accuracy of fine needle aspiration biopsy of thyroid nodules in detecting malignancy in childhood: comparison with conventional clinical, laboratory, and imaging approaches. J Clin Endocrinol Metab.

[37] Viswanathan K, Gierlowski TC, Schneider AB (1994). Childhood thyroid cancer. Characteristics and long-term outcome in children irradiated for benign conditions of the head and neck. Arch Pediatr Adolesc Med.

[38] Chiu HK, Sanda S, Fechner PY, Pihoker C (2012). Correlation of TSH with the risk of paediatric thyroid carcinoma. Clin Endocrinol (Oxf).

[39] Fiore E, Vitti P (2012). Serum TSH and risk of papillary thyroid cancer in nodular thyroid disease. J Clin Endocrinol Metab.

[40] Fiore E, Rago T, Provenzale MA, Scutari M, Ugolini C, Basolo F, Coscio G, Berti P, Grasso L, Elisei R, Pinchera A, Vitti P (2009). Lower levels of TSH are associated with a lower risk of papillary thyroid cancer in patients with thyroid nodular disease: thyroid autonomy may play a protective role. Endocr Relat Cancer.

[41] Fiore E, Rago T, Provenzale MA, Scutari M, Ugolini C, Basolo F, Coscio G, Miccoli P, Grasso L, Pinchera A, Vitti P (2010). L-thyroxine treated patients with nodular goiter have lower serum TSH and lower frequency of papillary thyroid cancer: results of a cross sectional study on 27 914 patients. Endocr Relat Cancer.

[42] Mondal HP, Sen S, Sasmal S, Ghosal PK, Mukhopadhyay SK, Mukhopadhyay M (2011). Clinicopathological correlation of serum TSH in patients with thyroid nodule. J Indian Med Assoc.

[43] Haymart MR, Repplinger DJ, Leverson GE, Elson DF, Sippel RS, Jaume JC, Chen H (2008). Higher serum thyroid stimulating hormone level in thyroid nodule patients is associated with greater risks of differentiated thyroid cancer and advanced tumor stage. J Clin Endocrinol Metab.

[44] Boelaert K (2009). The association between serum TSH concentration and thyroid cancer. Endocr Relat Cancer.

[45] Tonacchera M, Pinchera A, Vitti P (2010). Assessment of nodular goitre. Best Pract Res Clin Endocrinol Metab.

[46] Cooper DS, Doherty GM, Haugen BR, Kloos RT, Lee SL, Mandel SJ, Mazzaferri EL, McIver B, Pacini F, Schlumberger M, Sherman SI, Steward DL, Tuttle RM (2009). Revised American Thyroid Association management guidelines for patients with thyroid nodules and differentiated thyroid cancer. Thyroid.

[47] Daniels GH (2011). Screening for medullary thyroid carcinoma with serum calcitonin measurements in patients with thyroid nodules in the United States and Canada. Thyroid.

[48] Costante G, Meringolo D, Durante C, Bianchi D, Nocera M, Tumino S, Crocetti U, Attard M, Maranghi M, Torlontano M, Filetti S (2007). Predictive value of serum calcitonin levels for preoperative diagnosis of medullary thyroid carcinoma in a cohort of 5817 consecutive patients with thyroid nodules. J Clin Endocrinol Metab.

[49] Pacini F, Schlumberger M, Dralle H, Elisei R, Smit JW (2006). European consensus for the management of patients with differentiated thyroid carcinoma of the follicular epithelium.. Eur J Endocrinol.

[50] Costante G, Filetti S (2011). Early diagnosis of medullary thyroid carcinoma: is systematic calcitonin screening appropriate in patients with nodular thyroid disease?. Oncologist.

[51] Gharib H, Papini E, Paschke R, Duick DS, Valcavi R, Hegedus L (2010). American Association of Clinical Endocrinologists, Associazione Medici Endocrinologi, and European Thyroid Association Medical guidelines for clinical practice for the diagnosis and management of thyroid nodules: executive summary of recommendations. Endocr Pract.

[52] Elisei R (2008). Routine serum calcitonin measurement in the evaluation of thyroid nodules. Best Pract Res Clin Endocrinol Metab.

[53] Massaro F, Dolcino M, Degrandi R, Ferone D, Mussap M, Minuto F, Giusti M (2009). Calcitonin assay in wash-out fluid after fine-needle aspiration biopsy in patients with a thyroid nodule and border-line value of the hormone. J Endocrinol Invest.

[54] Costante G, Durante C, Francis Z, Schlumberger M, Filetti S (2009). Determination of calcitonin levels in C-cell disease: clinical interest and potential pitfalls. Nat Clin Pract Endocrinol Metab.

[55] Kudo T, Miyauchi A, Ito Y, Takamura Y, Amino N, Hirokawa M (2007). Diagnosis of medullary thyroid carcinoma by calcitonin measurement in fine-needle aspiration biopsy specimens. Thyroid.

[56] Goldfarb M, Gondek SS, Lew JI. (2012). Clinic-based ultrasound can predict malignancy in pediatric thyroid nodules.. Thyroid.

[57] Yoon JH, Kwak JY, Moon HJ, Kim MJ, Kim EK (2011). The diagnostic accuracy of ultrasound-guided fine-needle aspiration biopsy and the sonographic differences between benign and malignant thyroid nodules 3 cm or larger. Thyroid.

[58] Kabaker AS, Tublin ME, Nikiforov YE, Armstrong MJ, Hodak SP, Stang MT, McCoy KL, Carty SE, Yip L. (2012). Suspicious Ultrasound Characteristics Predict BRAF V600E-Positive Papillary Thyroid Carcinoma.. Thyroid.

[59] Mazzaferri EL (1990). Thyrotoxicosis. Results and risks of current therapy. Postgrad Med.

[60] Corrias A, Mussa A, Wasniewska M, Segni M, Cassio A, Salerno M, Gastaldi R, Vigone MC, Bal M, Matarazzo P, Weber G, De Luca F (2011). Levothyroxine treatment in pediatric benign thyroid nodules. Horm Res Paediatr.

[61] Chang YW, Hong HS, Choi DL (2009). Sonography of the pediatric thyroid: a pictorial essay. J Clin Ultrasound.

[62] Babcock DS (2006). Thyroid disease in the pediatric patient: emphasizing imaging with sonography. Pediatr Radiol.

[63] Rago T, Vitti P (2008). Role of thyroid ultrasound in the diagnostic evaluation of thyroid nodules. Best Pract Res Clin Endocrinol Metab.

[64] Rago T, Scutari M, Santini F, Loiacono V, Piaggi P, Coscio G, Basolo F, Berti P, Pinchera A, Vitti P (2010). Real-time elastosonography: useful tool for refining the presurgical diagnosis in thyroid nodules with indeterminate or nondiagnostic cytology. J Clin Endocrinol Metab.

[65] Stevens C, Lee JK, Sadatsafavi M, Blair GK (2009). Pediatric thyroid fine-needle aspiration cytology: a meta-analysis. J Pediatr Surg.

[66] Roy R, Kouniavsky G, Schneider E, Allendorf JD, Chabot JA, Logerfo P, Dackiw AP, Colombani P, Zeiger MA, Lee JA (2011). Predictive factors of malignancy in pediatric thyroid nodules. Surgery.

[67] Altincik A, Demir K, Abaci A, Bober E, Buyukgebiz A (2010). Fine-needle aspiration biopsy in the diagnosis and follow-up of thyroid nodules in childhood. J Clin Res Pediatr Endocrinol.

[68] Wiersinga WM (2007). Management of thyroid nodules in children and adolescents.. Hormones (Athens).

[69] Dinauer C, Francis GL (2007). Thyroid cancer in children. Endocrinol Metab Clin North Am.

[70] Gharib H (1997). Changing concepts in the diagnosis and management of thyroid nodules. Endocrinol Metab Clin North Am.

[71] Gutman PD, Henry M (1998). Fine needle aspiration cytology of the thyroid. Clin Lab Med.

[72] Hung W (1999). Solitary thyroid nodules in 93 children and adolescents. a 35-years experience. Horm Res.

[73] Degnan BM, McClellan DR, Francis GL (1996). An analysis of fine- needle aspiration biopsy of the thyroid in children and adolescents. J Pediatr Surg.

[74] Feinmesser R, Lubin E, Segal K, Noyek A (1997). Carcinoma of the thyroid in children-a review. J Pediatr Endocrinol Metab.

[75] Gharib H, Mazzaferri EL (1998). Thyroxine suppressive therapy in patients with nodular thyroid disease. Ann Intern Med.

[76] Giuffrida D, Gharib H (1995). Controversies in the management of cold, hot, and occult thyroid nodules. Am J Med.

[77] Papini E, Guglielmi R, Bianchini A, Crescenzi A, Taccogna S, Nardi F, Panunzi C, Rinaldi R, Toscano V, Pacella CM (2002). Risk of malignancy in nonpalpable thyroid nodules: predictive value of ultrasound and color-Doppler features. J Clin Endocrinol Metab.

[78] Gandolfi PP, Frisina A, Raffa M, Renda F, Rocchetti O, Ruggeri C, Tombolini A (2004). The incidence of thyroid carcinoma in multinodular goiter: retrospective analysis. Acta Biomed.

[79] Papotti M, Volante M, Saggiorato E, Deandreis D, Veltri A, Orlandi F (2002). Role of galectin-3 immunodetection in the cytological diagnosis of thyroid cystic papillary carcinoma. Eur J Endocrinol.

[80] Hung W, Sarlis NJ (2002). Current controversies in the management of pediatric patients with well-differentiated nonmedullary thyroid cancer: a review. Thyroid.

[81] Orlandi A, Puscar A, Capriata E, Fideleff H (2005). Repeated fine-needle aspiration of the thyroid in benign nodular thyroid disease: critical evaluation of long-term follow-up. Thyroid.

[82] Castro MR, Gharib H (2005). Continuing controversies in the management of thyroid nodules. Ann Intern Med.

[83] Sclabas GM, Staerkel GA, Shapiro SE, Fornage BD, Sherman SI, Vassillopoulou-Sellin R, Lee JE, Evans DB (2003). Fine-needle aspiration of the thyroid and correlation with histopathology in a contemporary series of 240 patients. Am J Surg.

[84] Mijovic T, Rochon L, Gologan O, Hier MP, Black MJ, Young J, Payne RJ (2009). Fine-needle aspiration biopsies in the management of indeterminate follicular and Hurthle cell thyroid lesions. Otolaryngol Head Neck Surg.

[85] Mehanna HM, Jain A, Morton RP, Watkinson J, Shaha A. (2009). Investigating the thyroid nodule.. BMJ.

[86] Unluturk U, Erdogan MF, Demir O, Gullu S, Baskal N (2012). Ultrasound elastography is not superior to grayscale ultrasound in predicting malignancy in thyroid nodules. Thyroid.

[87] Lippolis PV, Tognini S, Materazzi G, Polini A, Mancini R, Ambrosini CE, Dardano A, Basolo F, Seccia M, Miccoli P, Monzani F (2011). Is elastography actually useful in the presurgical selection of thyroid nodules with indeterminate cytology?. J Clin Endocrinol Metab.

[88] Saggiorato E, De Pompa R, Volante M, Cappia S, Arecco F, Dei Tos AP, Orlandi F, Papotti M (2005). Characterization of thyroid 'follicular neoplasms' in fine-needle aspiration cytological specimens using a panel of immunohistochemical markers: a proposal for clinical application. Endocr Relat Cancer.

[89] Raggio E, Camandona M, Solerio D, Martino P, Franchello A, Orlandi F, Gasparri G (2009). The diagnostic accuracy of the immunohistochemical markers in the preoperative evaluation of follicular thyroid lesions. J Endocrinol Invest.

[90] Saggiorato E, Cappia S, De Giuli P, Mussa A, Pancani G, Caraci P, Angeli A, Orlandi F (2001). Galectin-3 as a presurgical immunocytodiagnostic marker of minimally invasive follicular thyroid carcinoma. J Clin Endocrinol Metab.

[91] Cochand-Priollet B, Dahan H, Laloi-Michelin M, Polivka M, Saada M, Herman P, Guillausseau PJ, Hamzi L, Pote N, Sarfati E, Wassef M, Combe H, Raulic-Raimond D, Chedin P, Medeau V, Casanova D, Kania R (2011). Immunocytochemistry with cytokeratin 19 and anti-human mesothelial cell antibody (HBME1) increases the diagnostic accuracy of thyroid fine-needle aspirations: preliminary report of 150 liquid-based fine-needle aspirations with histological control. Thyroid.

[92] Nikiforov YE, Ohori NP, Hodak SP, Carty SE, LeBeau SO, Ferris RL, Yip L, Seethala RR, Tublin ME, Stang MT, Coyne C, Johnson JT, Stewart AF, Nikiforova MN (2011). Impact of mutational testing on the diagnosis and management of patients with cytologically indeterminate thyroid nodules: a prospective analysis of 1056 FNA samples. J Clin Endocrinol Metab.

[93] Li H, Robinson KA, Anton B, Saldanha IJ, Ladenson PW (2011). Cost-effectiveness of a novel molecular test for cytologically indeterminate thyroid nodules. J Clin Endocrinol Metab.

[94] Yip L, Farris C, Kabaker AS, Hodak SP, Nikiforova MN, McCoy KL, Stang MT, Smith KJ, Nikiforov YE, Carty SE (2012). Cost impact of molecular testing for indeterminate thyroid nodule fine-needle aspiration biopsies. J Clin Endocrinol Metab.

[95] Deandreis D, Al Ghuzlan A, Auperin A, Vielh P, Caillou B, Chami L, Lumbroso J, Travagli JP, Hartl D, Baudin E, Schlumberger M, Leboulleux S (2012). Is (18)F-fluorodeoxyglucose-PET/CT useful for the presurgical characterization of thyroid nodules with indeterminate fine needle aspiration cytology?. Thyroid.

